# Identification and characterization of bile microbiota in patients with biliary obstructive diseases using next-generation sequencing of 16S rRNA and ITS

**DOI:** 10.3389/fcimb.2025.1575824

**Published:** 2025-04-07

**Authors:** Jie Wang, Yu Wang, Jin Cao, Yong Chen, Juan Yu, Ning Sun

**Affiliations:** ^1^ Clinical Medicine Research Center, The Affiliated Suqian First People’s Hospital of Nanjing Medical University, Suqian, China; ^2^ Department of Clinical Laboratory Science, Jinling Hospital, Affiliated Hospital of Medical School, Nanjing University, Nanjing, China; ^3^ Department of Clinical Laboratory, Nanjing Lishui People’s Hospital, Nanjing, China

**Keywords:** biliary obstructive diseases, bile microbiome, 16S rRNA, ITS, next-generation sequencing, infection

## Abstract

**Background:**

Comparative studies of the bile microbiota in different biliary obstructive infections remain limited. This study aims to characterize bile microbiota and investigate differences in microbial profiles across various biliary obstructive diseases.

**Methods:**

This study included patients with biliary obstructive diseases admitted to Jinling Hospital and Suqian First Hospital. The cohort consisted of individuals with benign biliary disorders, malignant biliary obstruction, and biliary obstruction secondary to severe acute pancreatitis (SAP) or intestinal fistulas. A total of 133 bile samples were collected from 118 patients and analyzed using next-generation sequencing (NGS) targeting the bacterial 16S rRNA gene and the fungal internal transcribed spacer (ITS) gene. Clinical data, including routine culture results, were extracted from electronic medical records.

**Results:**

NGS targeting the 16S rRNA and ITS revealed a positive rate of 68.42% for bile samples, which was higher than the culture positivity rate of 60.15%, indicating a significant difference (Chi-square test, *p* < 0.05). The predominant bacteria identified in the bile samples through NGS were *Klebsiella pneumoniae*, *Acinetobacter baumannii*, and *Escherichia coli*. Bacterial species varied among benign biliary diseases, malignant obstructive diseases, and biliary obstruction caused by SAP or intestinal fistulas. Fungi were detected in 7.52% (10/133) of the samples, with 4 samples obtained from patients with biliary obstructive diseases due to SAP. Microbial diversity and clustering analysis showed no significant differences among various biliary disorders. Based on the culture results, the sensitivity and specificity of NGS were 81.82% and 69.64%, respectively.

**Conclusion:**

The composition of bile microbes may be related to the etiology of biliary obstruction. *Klebsiella pneumoniae*, *Acinetobacter baumannii*, and *Escherichia coli* are the predominant bacteria found in bile. NGS can be effectively applied for the identification and characterization of bile microbes associated with various biliary obstruction diseases.

## Introduction

In healthy individuals, bile is generally regarded as a harsh environment for microbial growth due to the flushing effect and the bacteriostatic properties of bile acids (Hofmann and Eckmann, 2006; Park et al., 2014; Watanabe et al., 2022). However, biliary obstruction disrupts the natural defense mechanisms, thereby allowing bacteria to invade biliary tract through either ascending infection from the duodenum or hematogenous spread through the hepatic portal vein (Chandra et al., 2019; Shafagh et al., 2021). Importantly, bile microbiota often play a crucial role in the pathogenesis and progression of these disorders, potentially leading to increased inflammation, sepsis, and life-threatening complications (Melzer et al., 2007). Numerous biliary obstructive diseases are associated with biliary tract infections (BTIs), including cholecystitis (Dyrhovden et al., 2020; Yan et al., 2021), choledocholithiasis (Han, 2021), cholangitis (Chandra et al., 2019) and other malignant tumors as well as pancreatitis (Wang et al., 2021). Therefore, a thorough analysis of bacterial infections in biliary disorders is crucial for initiating effective antimicrobial therapy.

In a clinical microbiological laboratory, culture is an indispensable and routine method for the detection and identification of bile bacteria (Kwon et al., 2013; Liu et al., 2024). However, culture-based methods may fail to accurately identify low-abundance, fastidious, and/or anaerobic bacteria, potentially leading to an incomplete understanding of the microbial composition. In most studies, *Escherichia coli* and other intestinal bacteria, such as *Klebsiella pneumoniae*, and *Enterococcus* are the most common isolates found in bile (Kwon et al., 2013; Dyrhovden et al., 2020; Wang et al., 2021; Yan et al., 2021; Asukai et al., 2023; Lee et al., 2023). Compared to traditional culture methods, NGS targeting the bacterial 16S rRNA gene (16S rRNA NGS) and the fungal internal transcribed spacer (ITS NGS) gene has become a standard approach in microbiome analysis across various environments (Nilsson et al., 2009; Dyrhovden et al., 2020; Yan et al., 2021; Wensel et al., 2022; Sun et al., 2023). Previous studies have focused on the role of bile microbiota in gallstone formation, the microbial composition in acute cholecystitis (Dyrhovden et al., 2020; Lee et al., 2023), cholelithiasis (Petrov et al., 2020; Hu et al., 2023), primary biliary cholangitis (Özdirik et al., 2023), and liver transplantation (Pérez-Cameo et al., 2020; D’Amico et al., 2021). However, investigations comparing bile microbial profiles among various biliary obstructive diseases remain limited. Biliary obstruction can result from diverse etiologies, including cholangiocarcinoma, pancreatic cancer, choledocholithiasis, and benign strictures, each of which may shape distinct microbial communities in bile. Understanding these potential differences is crucial, as they could provide insights into disease-specific pathogenesis. For example, in gastrointestinal diseases such as severe acute pancreatitis (SAP) and intestinal fistula, biliary obstruction is a common complication that requires decompression of the obstructed bile duct by percutaneous or perioperative drainage (Isayama et al., 2024). Moreover, bile microbiota may serve as an important source of pancreatic or abdominal infection, representing a considerable risk factor for acute biliary pancreatitis (Ni et al., 2023).

This study aims to analyze the composition of the bile microbiome and elucidate the species distribution using NGS targeting the 16S rRNA and ITS genes, providing new insights into the role of bile microbiota in these diseases and potentially offering novel strategies for diagnosing and treating biliary infections.

## Materials and methods

### Patients and clinical specimens

This study included 118 patients with biliary obstruction, from whom 133 bile samples were collected at Jinling Hospital and Suqian First Hospital between April and October 2023. These samples originated from the residual portions of routine microbiological tests conducted on patients with biliary obstruction following surgeries, including endoscopic retrograde cholangiopancreatography (ERCP; a procedure to diagnose and treat bile duct and pancreatic duct disorders), percutaneous transhepatic cholangial drainage (PTCD; a method to relieve bile duct obstruction by inserting a drainage tube through the liver), percutaneous cholecystostomy (a minimally invasive procedure to drain the gallbladder), or cholecystectomy (surgical removal of the gallbladder). The samples were immediately sent to the microbiology laboratory for testing after surgery, and upon completion of the tests, the remaining samples were stored at -80 °C. This study was approved by the Ethics Committees of Jinling Hospital (2021DZGZR-YBB-013) and Suqian First People’s Hospital (2023-SL-0049). Written informed consent was obtained from all participants or from their legal guardian/next of kin.

### Routine culture

As described in our previous study (Sun et al., 2023), a drop of bile was collected for smear microscopy, and the bile sample was then cultured aerobically on blood agar, chocolate agar, and MacConkey agar (Thermo Fisher Scientific Inc., Shanghai, China), respectively. The plates were incubated at 35°C. Chocolate agar plates, which are suitable for the growth of microaerophiles, were incubated in a 5% CO_2_ atmosphere, while the other plates were incubated without CO_2_. All plates were examined within 72 hours. A biochemical test was performed for the identification of bacteria and fungi using the VITEK^®^ 2 COMPACT (bioMérieux, Marcy-l’Étoile, France).

### Sample processing and DNA extraction

Bile samples were centrifuged at 12,000 rpm for 10 min. After centrifugation, a 250 μl mixture containing both the precipitate and supernatant was retained. This retained mixture was then processed by bead beating with 3-mm nickel beads. DNA was subsequently extracted using a Qiagen DNA Mini Kit (QIAGEN China (Shanghai) Co., Ltd, Shanghai, China), according to the manufacturer’s instructions. DNA concentration was determined by measuring the absorbance at 260/280 nm using a NanoDrop 2000 spectrophotometer (Thermo Fisher Scientific Inc., Waltham, MA, USA). The extracted DNA was stored at -20°C until library preparation.

### Library preparation and NGS

As described in our previous study (Sun et al., 2023), we performed NGS targeting the V3-V4 region of the 16S rRNA gene with some modifications. Briefly, we used universal primers (341F, 5′-CCTAYGGGRBGCASCAG-3′, and 806R, 5′-GGACTACNNGGGTATCTAAT-3′) with overhanging adapter sequences to amplify the V3-V4 region. The PCR was conducted using a thermal cycler (Axygen MaxyGene II, Axygen Scientific Inc., Union City, USA) with a 25-μl reaction mixture containing 0.2 μM of each primer, 1× Phusion Master Mix (Phusion^®^ High-Fidelity PCR Master Mix, New England Biolabs Inc., Ipswich, MA, USA), and 20 ng of DNA template. The PCR conditions included an initial denaturation at 98°C for 30 s; followed by 30 cycles of denaturation at 98°C for 10 s, annealing at 55°C for 30 s, and extension at 72°C for 30 s; and a final extension at 70°C for 10 min. We analyzed the PCR products using 1.5% agarose gel electrophoresis and purified them with the TaKaRa MiniBEST DNA Fragment Purification Kit (Takara Bio Inc., Dalian, China).

In the second-stage PCR, we added index adapters to the ends of the first-stage PCR products, followed by purification. The second-stage PCR was carried out using a thermal cycler (Axygen MaxyGene II, Axygen Scientific Inc., Union City, USA), with a 50-μl reaction mixture containing 1 μM of each index primer, 1× Phusion Master Mix (Phusion^®^ High-Fidelity PCR Master Mix, New England Biolabs Inc., Ipswich, MA, USA), and 10 μl of the first-stage PCR product. The PCR reaction conditions were as follows: initial denaturation at 98°C for 30 s; 10 cycles of denaturation at 98°C for 10 s, annealing at 60°C for 30 s, and extension at 72°C for 30 s; followed by a final extension at 72°C for 10 min. Finally, the second-stage PCR products were purified using the TaKaRa MiniBEST DNA Fragment Purification Kit (Takara Bio Inc., Dalian, China) and quantified using a Qubit@2.0 Fluorometer (Thermo Fisher Scientific Inc., Waltham, MA, USA) and an Agilent Bioanalyzer 2100 system (Agilent Technologies Inc., Santa Clara, CA, USA). The pooled library was diluted and sequenced using the NovaSeq platform (Illumina, San Diego, CA, USA) with 2 × 250 bp paired-end reads. According to a previous study (Nilsson et al., 2009), the library preparation for ITS NGS was similar to that for 16S rRNA NGS, involving two stages of PCR with universal primers (ITS2-F: GTGAATCATCGARTC; ITS2-R: TCCTCCGCTTATTGAT) and index primers. The only difference is that the annealing temperature in the first-stage PCR is set at 53°C.

### Data analysis

Raw data from 16S rRNA and ITS NGS were processed and filtered to remove low-quality reads using Fastp (Ver. 0.23.2) with default settings (Chen et al., 2018). Subsequently, the cleaned reads were merged and then clustered using Vsearch (Ver. 2.22.1) based on 100% similarity (Rognes et al., 2016). Reads were aligned and annotated with the Basic Local Alignment Search Tool (BLAST, version 2.12.0+) (Camacho et al., 2009) against the National Center for Biotechnology Information 16S rRNA (https://ftp.ncbi.nlm.nih.gov/blast/db/16S_ribosomal_RNA.tar.gz) and ITS (https://ftp.ncbi.nlm.nih.gov/blast/db/ITS_RefSeq_Fungi.tar.gz) databases. The alignment parameters were set as follows: -evalue 1e-5 -outfmt “7 std stitle” -perc_identity 97 -max_target_seqs 1. To simplify the complexity of species classification, facilitating the calculation of species read counts, only the best alignment result was selected.

### Statistics analysis

The data were analyzed using R language (Ver. 4.3.3). Cross-tabulation was used to evaluate the clinical performance of 16S rRNA and ITS NGS. Alpha diversity of each sample was analyzed using Shannon’s index, calculated using the vegan R packages (Ver. 2.6-6.1). Principal co-ordinates analysis (PCoA) was performed for comparison of bile microbiota in patients with various diseases. The significance level was set at p < 0.05. A chi-square test was utilized to evaluate the clinical performance of 16S rRNA and ITS NGS.

## Results

### Clinical characteristics of patients

The clinical characteristics of the 118 patients included in this study were summarized in **Table 1**. Overall, the mean age of the patients was 56.9 years, and 70% (79/118) of patients were male. Bile samples were collected from the perioperative period or through percutaneous drainage. Before the collection of specimens, 112 patients had undergone antibiotic treatment, and 10 patients had a history of biliary surgery.

**Table 1:** Demographic and clinical characteristics of all the patients.

Number of patients	118
Gender, male	79 (67%)
Mean age (SD; median; min–max), years	56.9 (17.4; 58; 26-95)
Gall bladder stone	36 (30.50%)
Bile duct stone	22 (18.64%)
Ongoing antibiotic therapy	112 (94.92%)
Disease ^a^	
Chronic cholecystitis	31 (26.27%)
SAP	27 (22.88%)
Acute cholecystitis	17 (14.41%)
Intestinal fistula	14 (11.86%)
Malignant biliary obstruction	14 (11.86%)
Cholecystolithiasis	6 (5.08%)
Acute cholangitis	4 (3.39%)
Others	5 (4.24%)
History of biliary tract surgery	10 (8.47%)
Comorbidities	
Diabetes	29 (24.58%)
Hypertension	56 (47.46%)
Chronic bronchitis	9 (7.63%)
Renal insufficiency	7 (5.93%)

a SAP, severe acute pancreatitis; Others include three patients with multiple injuries and two patients after intestinal surgery.

### Next-generation sequencing data analysis of 16S rRNA and ITS

A total of 133 bile samples, along with two blank control samples, underwent 16S rRNA NGS. Although a few samples did not exhibit the expected PCR products during the first-stage analysis using 1.5% agarose gel electrophoresis, we adhered to the protocol and proceeded with the second-stage PCR and NGS. After removing low-quality reads, the average number of clean reads was 220,593 (median: 267,798; range: 1,870–561,762).

For ITS NGS, the first-stage PCR products amplified using universal ITS primers were examined via 1.5% agarose gel electrophoresis. The results indicated that 10 samples yielded PCR products of the expected length, allowing for successful ITS NGS; the remaining samples were discontinued, while the two blank control samples were processed for ITS NGS. After filtering out low-quality ITS sequencing reads, the mean number of clean reads (excluding the blank controls) was 181,361 (range: 81,498–271,966; median: 182,871).

Clustering analysis with 100% similarity was conducted using Vsearch (Rognes et al., 2016), yielding a total of 253,648 operational taxonomic units (OTUs) from ITS NGS and 1,181,815 OTUs from 16S rRNA NGS. These OTUs were annotated using NCBI 16S rRNA and ITS datasets, revealing that successful classification of 152,021 OTUs for ITS and 1,141,774 OTUs for 16S rRNA. Based on the blank control results, a cut-off value of 100 reads was established for both 16S rRNA and ITS NGS detections. Species detected in the blank controls were considered contaminants, and only species with reads in samples exceeding five times the corresponding reads in the blank controls were deemed non-contaminants.

### Bile microbes detected by 16S rRNA and ITS NGS with aerobic culture as a reference

**Figure 1:**
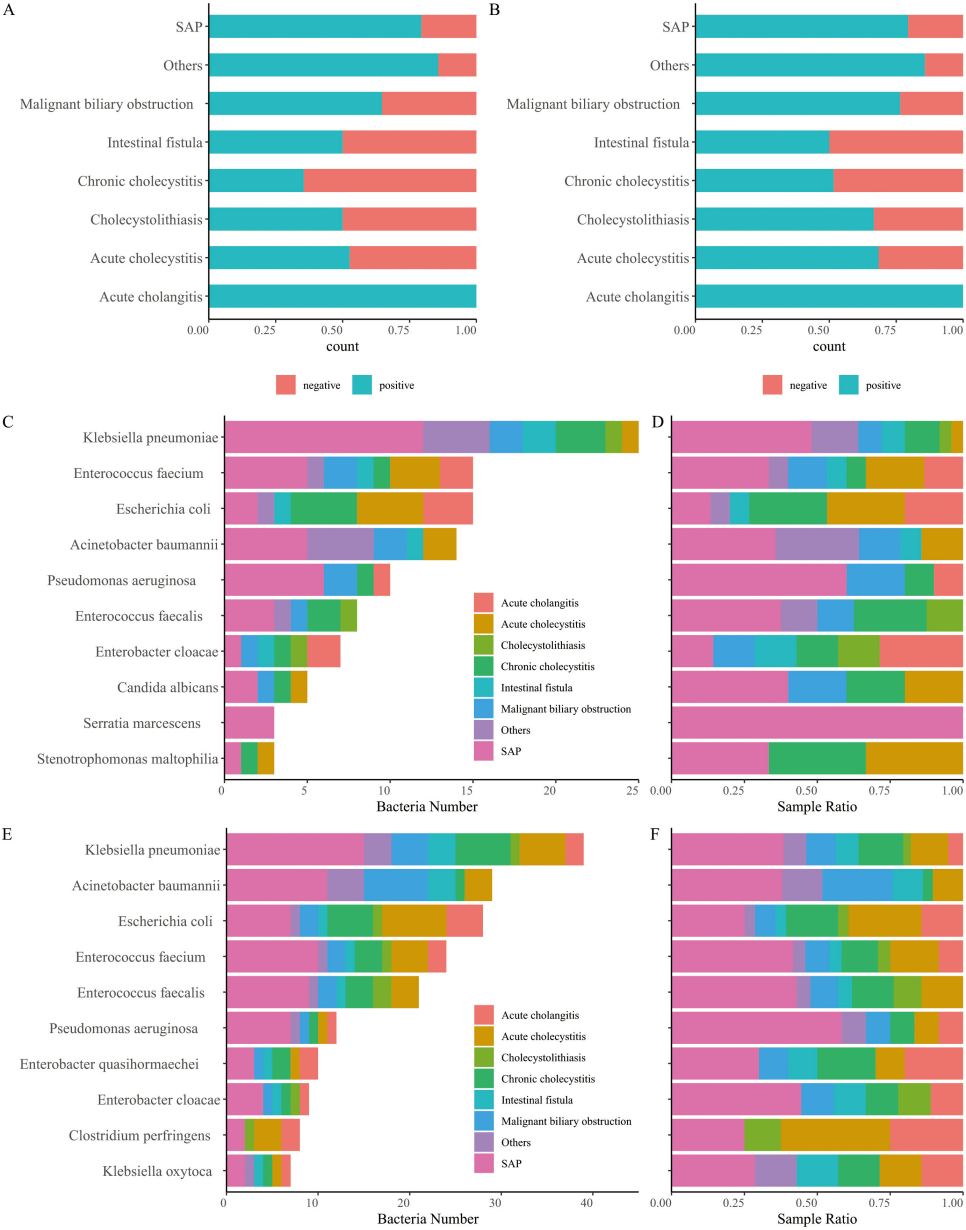
Comparison of positive and negative bile samples identified using culture **(A)** and next-generation sequencing (NGS) of 16S rRNA and ITS **(B)**. **(C)** Number of bacteria detected using culture. **(D)** Relative abundance of bacteria detected using culture. **(E)** Number of bacteria detected using 16S rRNA and ITS NGS. **(F)** Relative abundance of bacteria detected using 16S rRNA and ITS NGS.

The results of aerobic culture showed that 60.15% (80/133) of the samples were positive (**Figure 1A**), with the dominant species identified as *Klebsiella pneumoniae*, *Enterococcus faecium*, and *Escherichia coli* (**Figures 1C, D**; **Supplementary Table S1**). The positive rate of 16S rRNA and ITS NGS was 68.42% (91/133, **Figure 1B**), which was significantly higher than that of aerobic culture (Chi-square test, *p* < 0.05). Although there were few differences in dominant species between the culture and NGS results, the dominant species determined using 16S rRNA NGS were *K. pneumoniae*, *Acinetobacter baumannii*, and *E. coli* (**Figures 1E, F**; **Supplementary Table S1**). Additionally, we found that the microbial species in bile were broadly similar across various diseases. However, some differences were observed in bile bacteria for different biliary tract infections, regardless of the results obtained by culture or NGS (**Supplementary Figures S1**, **S2**). Notably, non-dominant species such as *Clostridium perfringens* and *Bacteroides fragilis* were detected in several samples using 16S rRNA NGS, particularly in severe infectious diseases such as SAP and intestinal fistulas, as well as in chronic cholecystitis. Importantly, we found that the positivity rate of bile varies, with the lowest positivity observed in chronic cholecystitis and choledocholithiasis, and the highest positivity recorded in acute cholangitis, or bile duct obstruction due to SAP or malignancy (**Figures 1A, B**).

### Difference of microbes in bile

**Figure 2:**
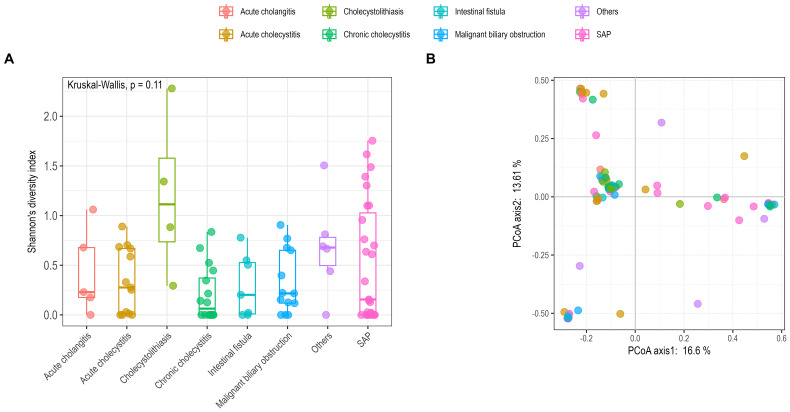
Comparison of alpha **(A)** and beta **(B)** diversity among different groups using Shannon’s diversity index and principal coordinate analysis (PCoA). **(A)** Alpha diversity was calculated using the R vegan package with the Shannon’s index; statistical analysis employed the Kruskal-Wallis test (*p* = 0.11). **(B)** Beta diversity was assessed based on Bray–Curtis dissimilarity with PCoA analysis; statistical analysis used Adonis2 (p < 0.05). SAP, severe acute pancreatitis.

All bile samples were classified into eight groups, SAP, intestinal fistula, Malignant biliary obstruction, acute cholangitis, acute cholecystitis, cholecystolithiasis, chronic cholecystitis, and others. Shannon’s diversity index was calculated to represent the alpha diversity of bile samples (**Figure 2A**). No significant difference was observed among the different groups (Kruskal-Wallis’s test, *p* > 0.05). Beta diversity among the different groups was significantly different (Adonis2, *p* < 0.05), although it only explains approximately 30% of the variance (**Figure 2B**; **Supplementary Figure S3**).

### Fungi detected by ITS NGS and culture

Fungal infections were detected in 7 out of 133 bile samples by culturing and in 10 samples by ITS NGS (**Table 2**). The results of the two methods were concordant in six samples; however, in one sample, culturing identified *Candida albicans*, while ITS NGS identified *Candida tropicalis*. ITS NGS also detected fungi in three samples that were culture-negative. Overall, ITS NGS detected fungi in 7.52% of bile samples (10/133), suggesting that the proportion of fungal presence in bile samples associated with biliary tract obstruction due to SAP may be higher.

**Table 2:** Fungi detected using culture and ITS NGS.

Sample ID	Disease	Culture	ITS NGS
D04	Malignant biliary obstruction	*Candida parapsilosis*	*Candida parapsilosis*
D19	Acute cholangitis	Undetermined	*Candida albicans*
D32	Choledocholithiasis	*Candida albicans*	*Candida tropicalis*
D33	Severe acute pancreatitis	*Candida albicans*	*Candida albicans*
D37	Severe acute pancreatitis	Undetermined	*Nakaseomyces glabratus*
D56	Gallbladder stone with chronic cholecystitis	*Candida albicans*	*Candida albicans*
D62	Intestinal fistula	*Candida parapsilosis*	*Candida parapsilosis*
D66	Malignant biliary obstruction	*Candida albicans*	*Candida albicans*
D85	Severe acute pancreatitis	Undetermined	*Candida albicans*
			*Nakaseomyces glabratus*
D88	Severe acute pancreatitis	*Candida albicans*	*Candida albicans*
			*Candida tropicalis*
			*Nakaseomyces glabratus*

### Clinical performance of *16S rRNA* and *ITS* NGS

Using culture as the reference standard, 16S rRNA and ITS NGS results were considered positive only when all cultured species were detected; otherwise, the results were considered negative. The clinical performance of *16S rRNA* and *ITS* NGS was then evaluated (**Table 3**). Compared to culture, the sensitivity and specificity were 81.82% and 69.64%, respectively, with an overall agreement of 76.69% (102/133).

**Table 3:** Clinical performance of *16S rRNA* and *ITS* NGS using culture as a reference ^a^.

		Culture								
		Positive	Negative	Sensitivity (%)	Specificity (%)	PPV (%)	NPV (%)	*p* value	Kappa	Agreement
16S rRNA and ITS NGS	Positive	63	14	81.82	69.64	78.75	73.58	< 0.001	0.518	76.69%
	Negative	17	39							

a Results of 16S rRNA and ITS NGS were considered positive only when all cultured species were detected; otherwise, results were considered negative. The Chi-square test revealed significant differences in microbe detection between culture and NGS (p < 0.001).

## Discussion

In this study, we analyzed 133 bile samples from 118 patients undergoing intraoperative or postoperative drainage for various biliary diseases. Among the patients, 36 (30.50%) had gallbladder stones and 22 (18.64%) had bile duct stones, both recognized risk factors for bile microbiota dysbiosis (Han, 2021; Lee et al., 2022; Sun et al., 2022). The distribution of biliary conditions reflected the severity of the disease, with chronic cholecystitis diagnosed in 31 patients (26.27%) and the more critical acute cholangitis in only 4 patients (3.39%). Our data suggest a higher positive rate in bile samples from critically ill patients, such as those with acute cholangitis and SAP, compared to patients with benign biliary diseases. This finding aligns with previous reports indicating a higher prevalence of pathogenic bacteria in critically ill individuals, underscoring the importance of incorporating bile microbiota analysis into clinical assessments of biliary diseases (Kwon et al., 2013; Dyrhovden et al., 2020; Binda et al., 2022).

Culture remains a primary method for clinical microbiological testing. However, anaerobic cultures are often not performed on bile samples due to practical limitations. Consequently, bile bacteria are primarily identified through aerobic culture, typically revealing predominant enteric bacteria such as *E. coli*, *K. pneumoniae*, *Enterococcus* spp (Lee et al., 2023; Chen et al., 2024). In our cohort, 16S rRNA NGS revealed that *K. pneumoniae* was the most frequent pathogen in bile from patients with SAP or chronic cholecystitis, while *E. coli* predominated in acute cholecystitis and acute cholangitis. These findings differ somewhat from previous culture-based studies (Maseda et al., 2013; Asukai et al., 2023). In patients with malignant biliary obstruction, *A. baumannii* was the most commonly found bacteria. Notably, 16S rRNA NGS also detected anaerobes such as *Clostridium perfringens*, and *Bacteroides fragilis*. The presence of Gram-positive bacteria like *Enterococcus faecium*, *Enterococcus faecalis*, and some isolates of *Streptococcus* spp. in bile was consistent with both culture results and previous study (Kwon et al., 2013; Dyrhovden et al., 2020; Poudel et al., 2023; Liu et al., 2024). Furthermore, ITS NGS detected fungi in 7.52% (10/133) of bile samples, with *Candida albicans* being the most frequent, followed by *Nakaseomyces glabratus*, *Candida parapsilosis*. These fungal detections were observed across a spectrum of diseases without significant difference.

Compared to benign biliary diseases (e.g., cholelithiasis, cholecystitis), malignant biliary diseases, SAP, intestinal fistula, and multiple injuries, can also cause biliary obstruction. Biliary drainage to reduce pressure is critical, as bile serves as a common clinical specimen for detecting pathogens in BTIs. While there are differences in bacterial distribution in bile across various diseases, a potential correlation exists between microbiome composition and disease severity. Notably, *K. pneumoniae*, a known pathogen in cholecystitis or cholangitis, was the most predominant pathogen in bile from SAP and intestinal fistulas. The composition of bile bacteria in SAP patients showed similarities to the bacterial profile of pancreatic infections in SAP patients from our previous study (Sun et al., 2023; Chen et al., 2025).

This study has several limitations. First, the sample size was relatively small, with a limited number of patients for some individual diseases, which may reduce the generalizability of our findings. Second, anaerobic culture was not performed due to laboratory constraints, potentially leading to an incomplete assessment of the anaerobic microbiota in bile. Third, there is no definitive method to validate NGS results and determine whether detected microorganisms are causative agents of infection or merely transient colonizers. Moreover, we acknowledge that the accuracy of 16S rRNA or ITS NGS results can be affected by factors such as DNA extraction quality, primer selection biases, and sequencing platform limitations. These technical challenges may introduce variability in the detection and quantification of microbial taxa. Additionally, the absence of healthy control samples limits our ability to determine whether observed microbial profiles represent pathological states or normal variations in bile microbiota. Finally, we recognize that the bioinformatics tools and pipelines employed for data analysis could significantly influence the interpretation of our results. The choice of algorithms and parameters may introduce biases, underscoring the need for transparent reporting and comparative analysis of bioinformatics approaches in microbiome analysis.

## Conclusion

In summary, this study utilized 16S rRNA and ITS NGS to characterize and identify the bile microbiota in patients with various biliary obstructive diseases. We found that *K. pneumoniae*, *A. baumannii*, and *E. coli* were the most dominant bacteria in bile, with notable similarities in microbial composition across different diseases, although some variations were observed for individual pathogens. The positivity rate of bile samples varied significantly, being highest in acute cholangitis and bile duct obstruction caused by SAP or malignancy, and lowest in chronic cholecystitis and choledocholithiasis. Fungal detection was also noted in a subset of samples, particularly those with SAP-related biliary obstruction. Compared to traditional culture methods, 16S rRNA and ITS NGS demonstrated higher sensitivity and specificity for bile microbe detection, with an agreement of 76.69%. These results indicate that NGS technologies can be effectively used to characterize the bile microbiome and correlate it with the type and severity of biliary disease, providing a more detailed understanding of the microbial landscape in biliary diseases.

## Data Availability

The datasets presented in this study are openly available in online repositories, specifically in the National Genomics Data Center at https://ngdc.cncb.ac.cn/gsub/submit/gsa/subCRA028124, reference number [PRJCA027619].

## Supplementary material

Supplementary Figure 1 Bile microbial profiles across different disease types as determined by culture.

Supplementary Figure 2 Bile microbial profiles in different disease types as determined by 16S rRNA and ITS NGS.

Supplementary Figure 3 Beta diversity analysis among different disease groups using principal coordinates analysis (PCoA) based on Bray-Curtis dissimilarity.

## References

[B1] Asukai K. Akita H. Mukai Y. Mikamori M. Hasegawa S. Fujii Y.. (2023). The utility of bile juice culture analysis for the management of postoperative infection after pancreaticoduodenectomy. Surgery 173, 1039–1044. doi: 10.1016/j.surg.2022.11.021 36549976

[B2] Binda C. Gibiino G. Coluccio C. Sbrancia M. Dajti E. Sinagra E.. (2022). Biliary diseases from the microbiome perspective: how microorganisms could change the approach to benign and Malignant diseases. Microorganisms 10, 312. doi: 10.3390/microorganisms10020312 35208765 PMC8877314

[B3] Camacho C. Coulouris G. Avagyan V. Ma N. Papadopoulos J. Bealer K.. (2009). BLAST+: architecture and applications. BMC Bioinf. 10, 421. doi: 10.1186/1471-2105-10-421 PMC2803857 20003500

[B4] Chandra S. Klair J. S. Soota K. Livorsi D. J. Johlin F. C. (2019). Endoscopic retrograde cholangio-pancreatography-obtained bile culture can guide antibiotic therapy in acute cholangitis. Dig. Dis. 37, 155–160. doi: 10.1159/000493579 30282078

[B5] Chen S. Lai W. Song X. Lu J. Liang J. Ouyang H.. (2024). The distribution and antibiotic-resistant characteristics and risk factors of pathogens associated with clinical biliary tract infection in humans. Front. Microbiol. 15. doi: 10.3389/fmicb.2024.1404366 PMC11112516 38784792

[B6] Chen S. Zhou Y. Chen Y. Gu J. (2018). fastp: an ultra-fast all-in-one FASTQ preprocessor. Bioinformatics 34, i884–i890. doi: 10.1093/bioinformatics/bty560 30423086 PMC6129281

[B7] Chen Y. Cui Q. Cao J. Wu Q. Lu P. Li G.. (2025). Characterization of pancreatic infections in patients with severe acute pancreatitis: A retrospective study from 2019 to 2023. Infect. Drug Resist. 18, 199–207. doi: 10.2147/IDR.S500916 39816241 PMC11734511

[B8] D’Amico F. Bertacco A. Finotti M. Di Renzo C. Rodriguez-Davalos M. I. Gondolesi G. E.. (2021). Bile microbiota in liver transplantation: proof of concept using gene amplification in a heterogeneous clinical scenario. Front. Surg. 8. doi: 10.3389/fsurg.2021.621525 PMC8009296 33796547

[B9] Dyrhovden R. Øvrebø K. K. Nordahl M. V. Nygaard R. M. Ulvestad E. Kommedal Ø. (2020). Bacteria and fungi in acute cholecystitis. A prospective study comparing next generation sequencing to culture. J. Infect. 80, 16–23. doi: 10.1016/j.jinf.2019.09.015 31586461

[B10] Han J. (2021). Biliary microbiota in choledocholithiasis and correlation with duodenal microbiota. Front. Cell. Infect. Microbiol. 11. doi: 10.3389/fcimb.2021.625589 PMC8116743 33996618

[B11] Hofmann A. F. Eckmann L. (2006). How bile acids confer gut mucosal protection against bacteria. Proc. Natl. Acad. Sci. U.S.A. 103, 4333–4334. doi: 10.1073/pnas.0600780103 16537368 PMC1450168

[B12] Hu J. Tang J. Zhang X. Yang K. Zhong A. Yang Q.. (2023). Landscape in the gallbladder mycobiome and bacteriome of patients undergoing cholelithiasis with chronic cholecystitis. Front. Microbiol. 14. doi: 10.3389/fmicb.2023.1131694 PMC10073429 37032855

[B13] Isayama H. Hamada T. Fujisawa T. Fukasawa M. Hara K. Irisawa A.. (2024). TOKYO criteria 2024 for the assessment of clinical outcomes of endoscopic biliary drainage. Dig. Endosc. 36, 1195–1210. doi: 10.1111/den.14825 38845085

[B14] Kwon W. Jang J.-Y. Kim E.-C. Park J. W. Han I. W. Kang M. J.. (2013). Changing trend in bile microbiology and antibiotic susceptibilities: over 12 years of experience. Infection 41, 93–102. doi: 10.1007/s15010-012-0358-y 23180506

[B15] Lee J. M. Kang J. S. Choi Y. J. Byun Y. Jin S. H. Yoon K. C.. (2023). Suggested use of empirical antibiotics in acute cholecystitis based on bile microbiology and antibiotic susceptibility. HPB 25, 568–576. doi: 10.1016/j.hpb.2023.01.017 36804057

[B16] Lee J. Park J.-S. Bae J. Lee S. Hwang Y. (2022). Bile microbiome in patients with recurrent common bile duct stones and correlation with the duodenal microbiome. Life 12, 1540. doi: 10.3390/life12101540 36294975 PMC9605223

[B17] Liu T. Li M. Tang L. Wang B. Li T. Huang Y.. (2024). Epidemiological, clinical and microbiological characteristics of patients with biliary tract diseases with positive bile culture in a tertiary hospital. BMC Infect. Dis. 24, 1010. doi: 10.1186/s12879-024-09799-8 39300331 PMC11414084

[B18] Maseda E. Maggi G. Gomez-Gil R. Ruiz G. Madero R. Garcia-Perea A.. (2013). Prevalence of and risk factors for biliary carriage of bacteria showing worrisome and unexpected resistance traits. J. Clin. Microbiol. 51, 518–521. doi: 10.1128/JCM.02469-12 23196362 PMC3553897

[B19] Melzer M. Toner R. Lacey S. Bettany E. Rait G. (2007). Biliary tract infection and bacteraemia: presentation, structural abnormalities, causative organisms and clinical outcomes. Postgrad. Med. J. 83, 773–776. doi: 10.1136/pgmj.2007.064683 18057178 PMC2750926

[B20] Ni T. Wen Y. Zhao B. Ning N. Chen E. Mao E.. (2023). Characteristics and risk factors for extrapancreatic infection in patients with moderate or severe acute pancreatitis. Heliyon 9, e13131. doi: 10.1016/j.heliyon.2023.e13131 36755607 PMC9900262

[B21] Nilsson R. H. Ryberg M. Abarenkov K. Sjökvist E. Kristiansson E. (2009). The ITS region as a target for characterization of fungal communities using emerging sequencing technologies. FEMS Microbiol. Lett. 296, 97–101. doi: 10.1111/j.1574-6968.2009.01618.x 19459974

[B22] Özdirik B. Scherf M. Brumercek A. Nicklaus J. M. Kruis T. Haber P. K.. (2023). Biliary microbial patterns in primary sclerosing cholangitis are linked to poorer transplant-free survival. Hepatol. Commun. 7, e0156. doi: 10.1097/HC9.0000000000000156 37204418 PMC10540062

[B23] Park J. W. Lee J. K. Lee K. T. Lee K. H. Sung Y. K. Kang C.-I. (2014). How to interpret the bile culture results of patients with biliary tract infections. Clinics Res. Hepatol. Gastroenterol. 38, 300–309. doi: 10.1016/j.clinre.2014.02.005 24674840

[B24] Pérez-Cameo C. Bilbao I. Lung M. Caralt M. Vargas V. Pont T.. (2020). Routine bile culture from liver donors as screening of donor-transmitted infections in liver transplantation. Liver Transpl. 26, 1121–1126. doi: 10.1002/lt.25778 32289870

[B25] Petrov V. A. Fernández-Peralbo M. A. Derks R. Knyazeva E. M. Merzlikin N. V. Sazonov A. E.. (2020). Biliary microbiota and bile acid composition in cholelithiasis. BioMed. Res. Int. 2020, 1–8. doi: 10.1155/2020/1242364 PMC7352139 32714973

[B26] Poudel S. K. Padmanabhan R. Dave H. Guinta K. Stevens T. Sanaka M. R.. (2023). Microbiomic profiles of bile in patients with benign and Malignant pancreaticobiliary disease. PloS One 18, e028302. doi: 10.1371/journal.pone.0283021 PMC10112786 37071646

[B27] Rognes T. Flouri T. Nichols B. Quince C. Mahé F. (2016). VSEARCH: a versatile open source tool for metagenomics. PeerJ 4, e2584. doi: 10.7717/peerj.2584 27781170 PMC5075697

[B28] Shafagh S. Rohani S. H. Hajian A. (2021). Biliary infection; distribution of species and antibiogram study. Ann. Med. Surg. 70, 102822. doi: 10.1016/j.amsu.2021.102822 PMC8435813 34540214

[B29] Sun N. Chen Y. Zhang J. Cao J. Huang H. Wang J.. (2023). Identification and characterization of pancreatic infections in severe and critical acute pancreatitis patients using 16S rRNA gene next generation sequencing. Front. Microbiol. 14. doi: 10.3389/fmicb.2023.1185216 PMC10303115 37389346

[B30] Sun H. Warren J. Yip J. Ji Y. Hao S. Han W.. (2022). Factors influencing gallstone formation: A review of the literature. Biomolecules 12, 550. doi: 10.3390/biom12040550 35454138 PMC9026518

[B31] Wang C. Yu H. He J. Li M. Zhang L. Xu Y.. (2021). Comparative analysis of bile culture and blood culture in patients with Malignant biliary obstruction complicated with biliary infection. J. Can. Res. Ther. 17, 726. doi: 10.4103/jcrt.JCRT_1705_20 34269306

[B32] Watanabe S. Minagawa M. Shinoda T. Motooka D. Tohya M. Kirikae T.. (2022). Bile collected from the normal gallbladder of patients during surgery has simple bacterial flora. Cureus 14, e25681. doi: 10.7759/cureus.25681 35812645 PMC9257430

[B33] Wensel C. R. Pluznick J. L. Salzberg S. L. Sears C. L. (2022). Next-generation sequencing: insights to advance clinical investigations of the microbiome. J. Clin. Invest. 132, e154944. doi: 10.1172/JCI154944 35362479 PMC8970668

[B34] Yan Q. Zhang S. Li S. Wang G. Zhang A. Jin T.. (2021). Cultivation and genomic characterization of the bile bacterial species from cholecystitis patients. Front. Microbiol. 12. doi: 10.3389/fmicb.2021.739621 PMC8591784 34790179

