# Supplementary figures and images for: Identification and characterization of bile microbiota in patients with biliary obstructive diseases using next-generation sequencing of 16S rRNA and ITS
Source: Front Cell Infect Microbiol. 2025 Apr 7;15:1575824. doi: 10.3389/fcimb.2025.1575824 (PMC12009824; doi:10.3389/fcimb.2025.1575824)

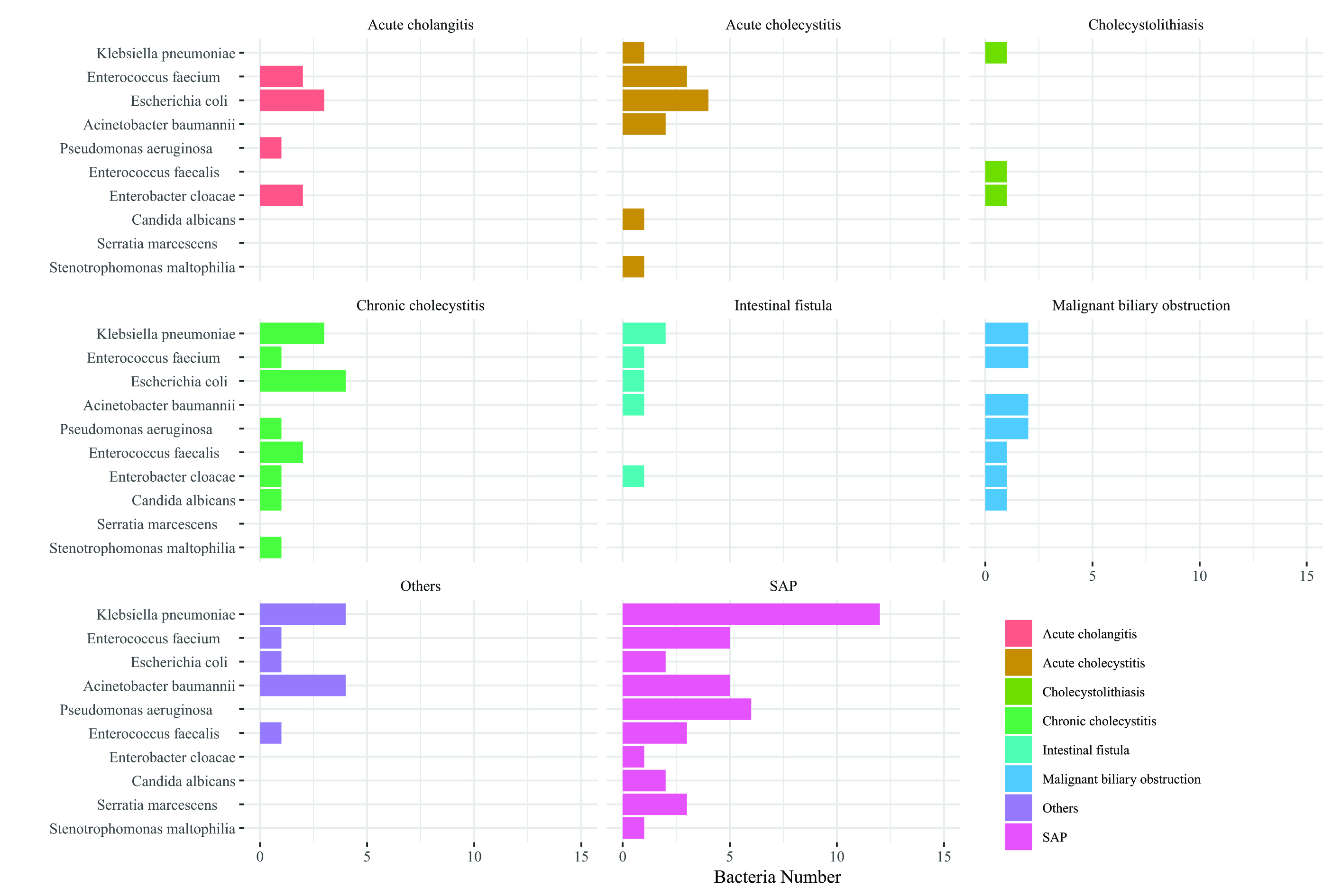

Supplement: Supplementary Figure 1 — Bile microbial profiles across different disease types as determined by culture. [file Image1.jpeg]

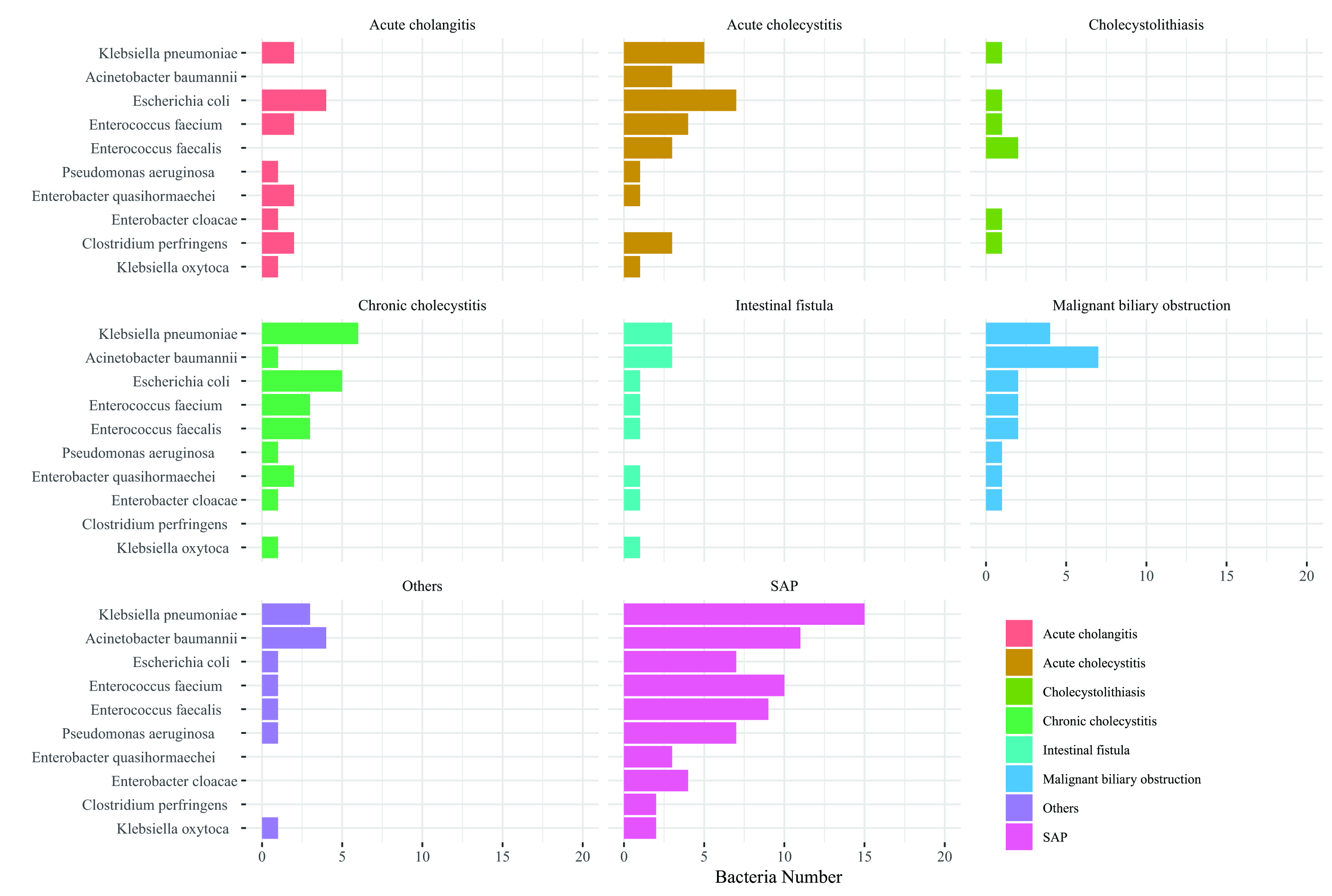

Supplement: Supplementary Figure 2 — Bile microbial profiles in different disease types as determined by 16S rRNA and ITS NGS. [file Image2.jpeg]

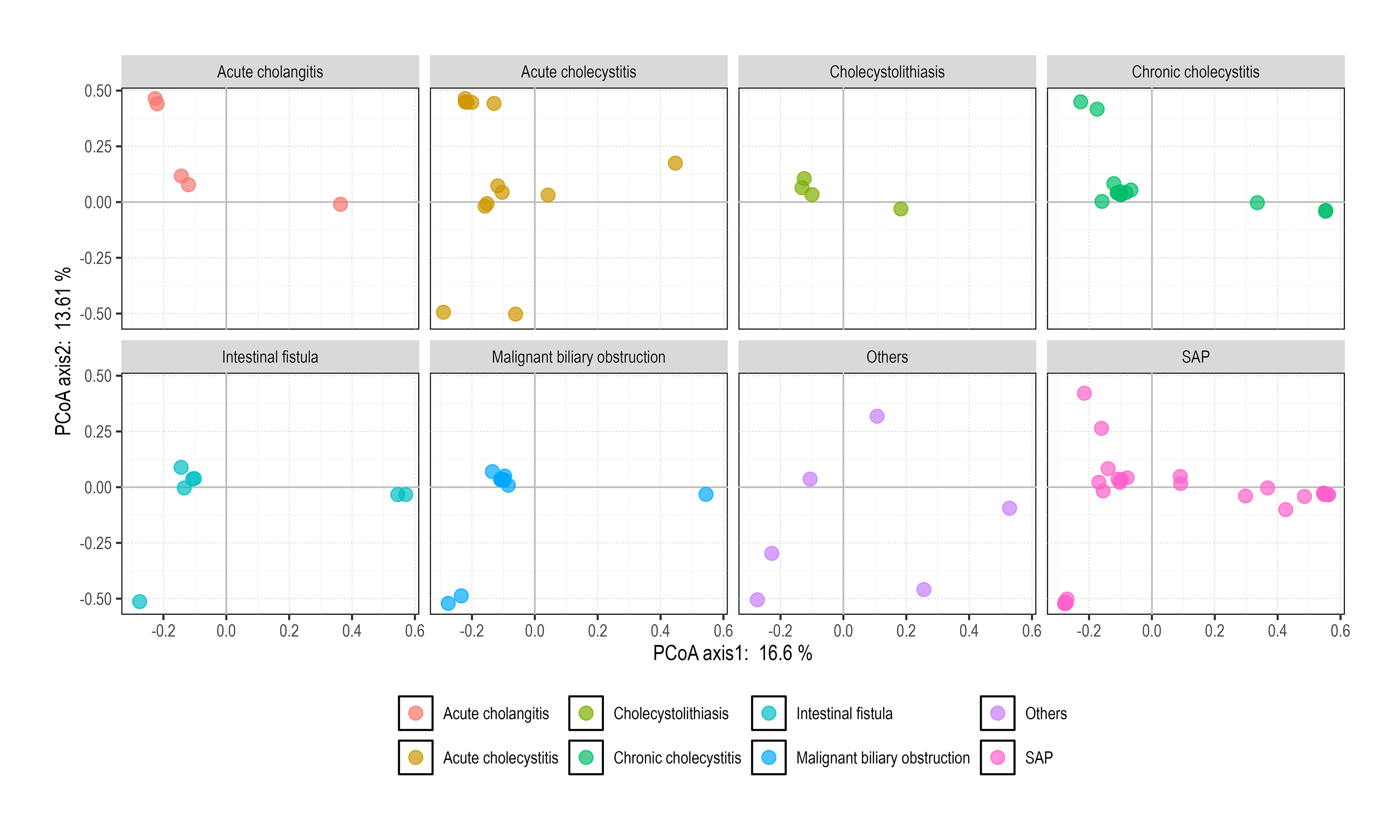

Supplement: Supplementary Figure 3 — Beta diversity analysis among different disease groups using principal coordinates analysis (PCoA) based on Bray-Curtis dissimilarity. [file Image3.jpeg]
